# Distinct selective forces and Neanderthal introgression shaped genetic diversity at genes involved in neurodevelopmental disorders

**DOI:** 10.1038/s41598-017-06440-4

**Published:** 2017-07-21

**Authors:** Alessandra Mozzi, Diego Forni, Rachele Cagliani, Uberto Pozzoli, Mario Clerici, Manuela Sironi

**Affiliations:** 1 Bioinformatics, Scientific Institute IRCCS E. MEDEA, 23842 Bosisio Parini, Italy; 2 Department of Physiopathology and Transplantation, University of Milan, 20090 Milan, Italy; 3 Don C. Gnocchi Foundation ONLUS, IRCCS, 20100 Milan, Italy

## Abstract

In addition to high intelligence, humans evolved specialized social-cognitive skills, which are specifically affected in children with autism spectrum disorder (ASD). Genes affected in ASD represent suitable candidates to study the evolution of human social cognition. We performed an evolutionary analysis on 68 genes associated to neurodevelopmental disorders; our data indicate that genetic diversity was shaped by distinct selective forces, including natural selection and introgression from archaic hominins. We discuss the possibility that segregation distortion during spermatogenesis accounts for a subset of ASD mutations. Finally, we detected modern-human-specific alleles in *DYRK1A* and *TCF4*. These variants are located within regions that display chromatin features typical of transcriptional enhancers in several brain areas, strongly suggesting a regulatory role. These SNPs thus represent candidates for association with neurodevelopmental disorders, and await experimental validation in future studies.

## Introduction

In nature, “intelligence” can be defined as the problem-solving ability to adapt to changes in natural and social environment. In the last decades, broad comparative analyses indicated that social interaction drives the evolution of higher cognitive abilities in animals^1, 2^, supporting the “social brain hypotesis”^3^. Although complex social systems are observed throughout the animal kingdom, vertebrates are considered to posses higher cognitive functions and more complex social behaviors than invertebrates^4, 5^. Recent studies suggest that vertebrate expansion in synapse proteome complexity, driven by the combined action of paralog diversification and alternative splicing, contributed to the behavioral and cognitive complexity of these species^6, 7^.

Among vertebrates, advanced cognitive abilities and complex behavioral patterns are observed in Mammalia and Aves. Within both classes, some lineages stand out for their higher cognition (e.g., primates, cetaceans, and elephants in mammals, corvids and parrots in birds)^8–10^. However, prosocial behaviors such as other-regarding preferences and reciprocity are thought to be more common in mammals compared to other vertebrates^11, 12^.

Among all animals, humans display the highest forms of intelligence, although uncertainty still exists about the timing of appearance of some cognitive and behavioral traits and on the sharing of these features with archaic extinct hominids^13, 14^. In modern humans, the evolution of cognitive functions led to the development of a grammatical and syntactical language, which has likely served as an intelligence amplifier^15^. Humans have also evolved specialized social-cognitive skills for living and exchanging knowledge in cultural groups^16^. Some of these skills are specifically affected in children with autism spectrum disorder (ASD). In fact, it was recently suggested^13^ that genes and cerebral circuits affected in ASD represent candidates for the evolution of human social cognition. Notably, it was proposed that the higher cognitive capacities in humans were acquired at the cost of increased susceptibility to mental disease^17–19^.

Psychiatric conditions such ASD and schizophrenia (SCZ) have a prevalence around 3–4% in human populations^20^. SCZ and ASD are characterized by a polygenic architecture and persist in populations despite a negative fitness effect. This apparent evolutionary paradox is explained by recent observations that, although common variants play a role in the pathogenesis of ASD and SCZ^21, 22^, rare or *de novo* mutations represent the major source of large-effect risk factors^23, 24^. Substantial overlap exists among genes mutated in ASD/SCZ and those associated with intellectual disability (ID)^24, 25^. ID has an overall prevalence of 1.5 to 2% in Western populations and is common in children diagnosed with ASD^25^. Overall, these observations suggest that the expansion of cognitive and social abilities also expanded the mutational target for ASD, SCZ, and ID, thus explaining their high prevalence.

Because of the huge genetic heterogeneity of these complex diseases, a reverse strategy based on the identification of networks of genes interconnected by a specific feature (e.g. biological function or protein-protein interactions) may help define genetic disease subtypes^26–28^. Starting from these assumptions, we analyzed the evolutionary history of two gene modules (M1 and M2), that are associated with different phenotypes and were previously identified by Hormozdiari and colleagues^29^. These modules were built through the exome sequencing data of 1116 patients affected by ASD and ID^29^. Module 1 includes genes associated to transcriptional regulation during brain development, whereas module 2 is composed of synaptic genes involved in long-term potentiation and calcium signaling^29^.

## Results

### Gene selection

**Figure 1:**
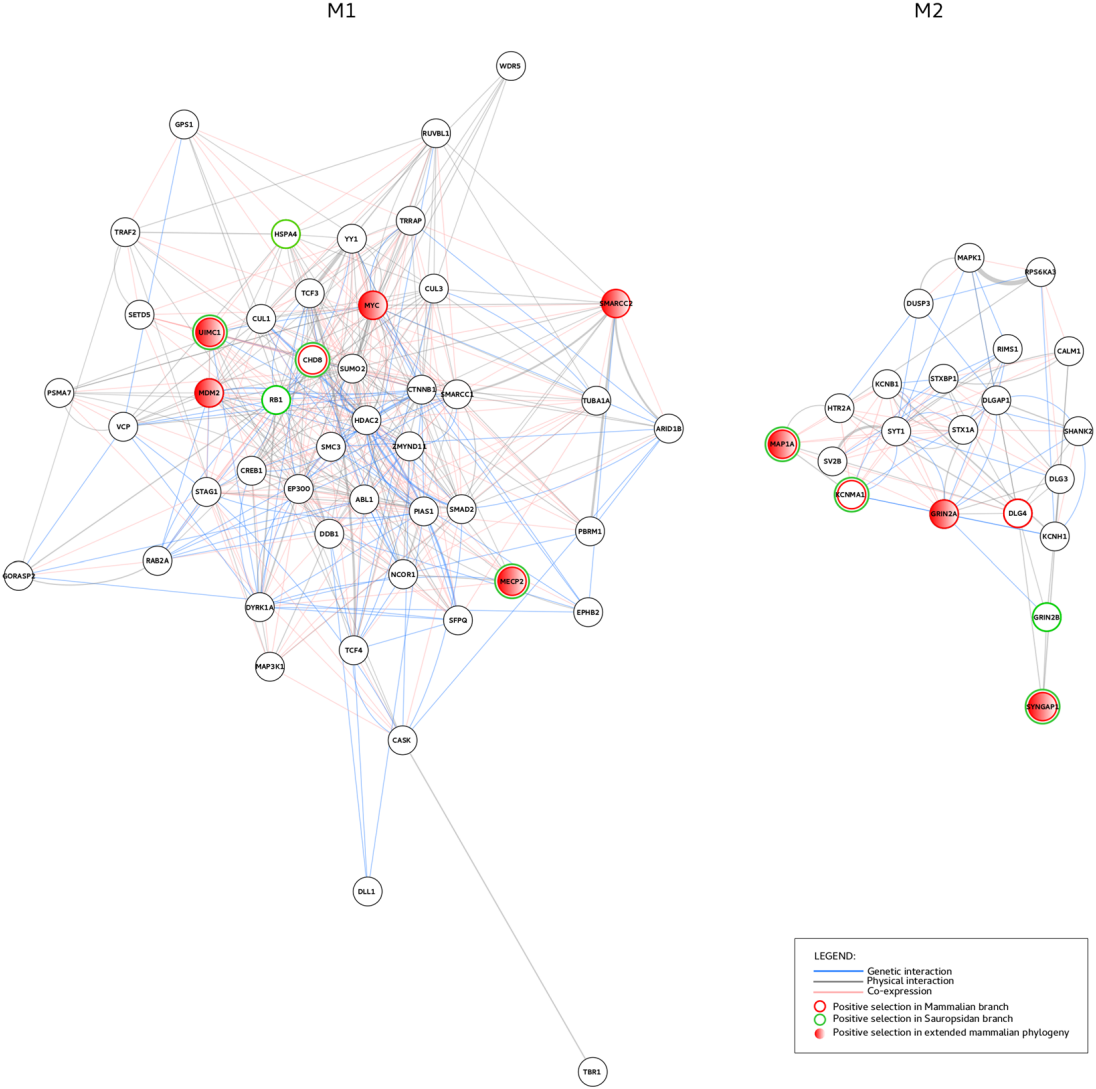
M1 and M2 gene networks. Interaction networks of module 1 (M1) an module 2 (M2) genes as obtained by Cytoscape, using the geneMANIA application.

We analyzed the evolutionary history of two different sets of genes (modules M1 and M2) previously identified by Hormozdiari and colleagues using MAGI (Merging Affected Genes into Integrated-networks)^29^. This computational method simultaneously integrates protein-protein interaction data, RNA expression profile, and the enrichment of *de novo* mutations in affected probands. Specifically, Hormozdiari and coworkers used expression data during brain development and *de novo* mutations from 6 studies of ASD and ID^29^. Each of these modules contains a subset of genes belonging to a common pathway. M1 consists of 47 genes associated with Wnt, Notch, SWI/SNF, and NCOR signaling, and showing high expression during embryonic development (8–16 pcw) (Fig. 1, Supplementary Table S1). M2 includes 21 genes associated with synaptic function and mainly expressed at postnatal stages (Fig. 1, Supplementary Table S1).

### Episodic positive selection in Mammalia and Sauropsida

**Figure 2:**
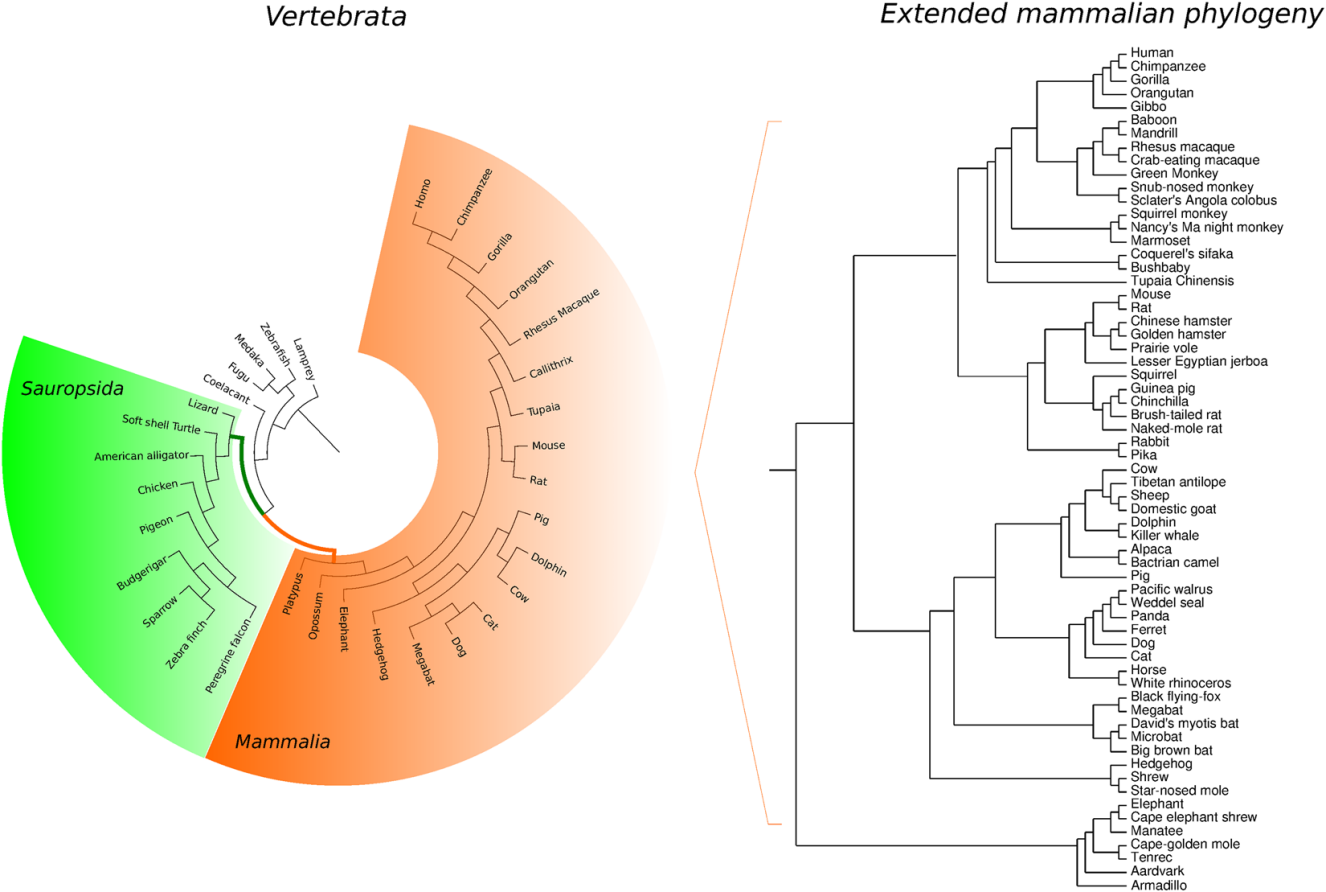
Vertebrata and mammalian phylogenies. Phylogenetic tree of the Vertebrata species used in our analyses. The mammalian and sauropsidan branches are highlighted in orange and green, respectively. The extended mammalian phylogeny is also reported on the right (Supplementary Table S2).

We first explored possible variations in selective pressure at M1 and M2 module genes among vertebrate species. In particular, we applied branch-site likelihood ratio tests (LRTs)^30^ to phylogenies that include representative vertebrate species from lamprey to human (Fig. 2, Supplementary Table S2). When recombination was detected, gene alignments were split on the basis of the recombination breakpoints.

LRTs were used to test the two internal branches of the phylogeny leading to the Mammalia and to the Sauropsida classes (Fig. 2). A false discovery rate (FDR) correction was applied, as suggested^31^. Positive selection was declared if neutral models were rejected in favor of the positive selection model using two codon frequency models (Table 1, Supplementary Table S3).

**Table 1:** Likelihood ratio test statistics for models of variable selective pressure along mammalian and sauropsidan branches (codon frequency:F3 × 4).

		*Mammalia*			*Sauropsida*		
		(*MA1−MA*) −*2ΔlnL* ^a^	*p* value^b^	MEME-BEB sites^c^	(*MA1−MA*) −*2ΔlnL* ^a^	*p* value^b^	MEME-BEB sites^c^
M1	*CHD8*	58.044	**2**.**56** **×** **10** ^−1**4**^	—	76.290	**4**.**90** **×** **10** ^−**18**^	D2199, D2487
	*HSPA4*	0	**1**	—	11.0829	**1**.**74** **×** **10** ^−**3**^	—
	*MDM2*	12.255	**9**.**28** **×** **10** ^−**4**^	L205	0	**1**	—
	*MECP2*	4.950	**2**.**61** **×** **10** ^−**2**^	—	21.963	**5**.**56** **×** **10** ^−**6**^	—
	*MYC*	10.527	**2**.**35** **×** **10** ^−**3**^	—	8.519	3.51 × 10^−3^	—
	*RB1*	0	**1**	—	15.493	**1**.**66** **×** **10** ^−**4**^	—
	*SMARCC2 Region 2* (*106–1152aa*)	15.721	**1**.**47** **×** **10** ^−**4**^	—	0	**1**	—
	*UIMC1*	7.634	**7**.**85** **×** **10** ^−**3**^	—	7.068	**7**.**85** **×** **10** ^−**3**^	—
M2	*DLG4*	5.320	**4**.**22** **×** **10** ^−**2**^	—	0	**1**	—
	*GRIN2A Region 3* (*867–1464aa*)	5.974	**1**.**73** **×** **10** ^−**2**^	G951, T1043	5.667	1.73 × 10^−2^	—
	*GRIN2B*	0	**1**	—	8.337	**7**.**77** **×** **10** ^−**3**^	—
	*KCNMA1*	18.850	**1**.**44** **×** **10** ^−**5**^	—	18.811	**1**.**44** **×** **10** ^−**5**^	C622
	*MAP1A*	61.374	**9**.**44** **×** **10** ^−**15**^	L610, S1029, G2436	32.349	**1**.**29** **×** **10** ^−**8**^	—
	*SYNGAP1 Region 2* (*115–1343aa*)	64.996	**1**.**50** **×** **10** ^−**15**^	R329, A1281	26.360	**2**.**83** **×** **10** ^−**7**^	—

^a^2ΔlnL: twice the difference of the natural logs of the maximum likelihood of the models being compared.

^b^ *p* values are FDR corrected.

^c^Positions refer to the human sequence (see Supplementary Table 1, Supplementary material).

Bolded *p*-values indicated LRTs confirmed by appling the F61 codon frequency model (see Supplementary Table 1, Supplementary material).

Evidence of positive selection was detected in 8/47 (17.0%) M1 genes and in 6/21 (28.6%) M2 genes (Table 1, Supplementary Table S3). Most genes with statistically-supported evidence of positive selection in mammals also showed evidence of selection in Sauropsida (Table 1, Supplementary Table S3). For instance, this was the case for *MECP2*, which causes Rett syndrome when mutated, and of *SYNGAP1*, whose mutations were associated with ID, ASD, and epilepsy^32, 33^. Three genes were only selected on the Sauropsida branch, and five only on the mammalian branch (Table 1). Overall, these data suggest that genes in the two modules were not targeted by stronger selective pressure in Mammalia compared to Sauropsida.

**Figure 3:**
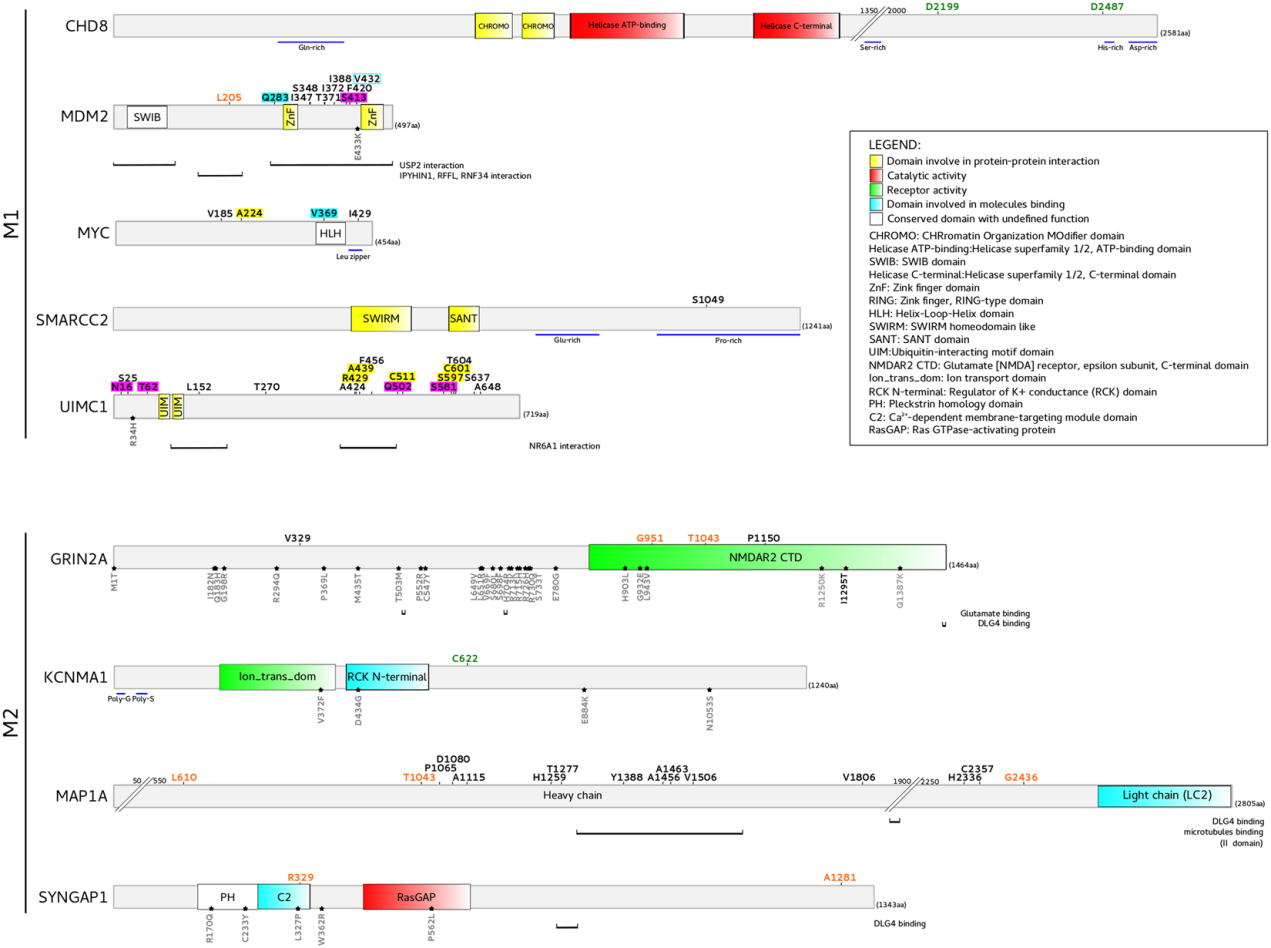
Domain representation of positively selected genes. Sites showing evidence of positive selection are mapped onto the domain representation of the proteins and colour-coded as follows: orange, positively selected sites on the mammalian branch; green, positively selected sites on the sauropsidan branch; black, positively selected sites in the mammalian phylogeny. Selected sites in the human, chimpanzee or gorilla lineages are highlighted in cyan, yellow and violet, respectively. Positions refer to human sequences (Supplementary Table S1). Domain names and functions are reported in the legend. Black stars indicate missense mutations associated to ASD, ID, SCZ, and other neurodevelopmental disorders^33, 79, 82, 120–123^.

Positively selected sites along the mammalian or sauropsidan branches were identified through the Bayes Empirical Bayes (BEB) analysis^30^. To be conservative, we considered as positively selected only sites also detected by the Mixed Effects Model of Evolution (MEME) method^34^. Using this criterion, a few positively selected sites were detected (Table 1, Fig. 3). Interestingly, three sites positively selected on the mammalian branch are located in two genes encoding major components of the postsynaptic density (PSD) of excitatory neuronal synapses: *GRIN2A* and *SYNGAP1*. In particular, two sites (G951 and T1043) map to the C-terminal domain (CTD) of GRIN2A, and one (R329) is located in the SYNGAP1 C2 domain, which is necessary for the Rap GTPase activity^35^ (Fig. 3).

### Positive selection across the mammalian phylogeny

The branch-site tests we applied in the section above are well-suited to search for episodic selection in a phylogeny of distantly related species^36^, but they are generally characterized by low statistical power^30^. Thus, for genes showing evidence of selection along the mammalian branch, we extended the evolutionary analysis to include additional species. The *codeml* site models^37^ were run on gene phylogenies of at least 60 mammalian species (Table 2, Supplementary Tables S2 and S4). When recombination was detected, gene alignments were split on the basis of the recombination breakpoints. Two neutral models (M8a and M7) were rejected in favor of the M8 positive selection model for the *MDM2*, *MECP2*, *MYC*, *SMARCC2*, and *UIMC1* genes in module 1, as well as for *GRIN2A*, *MAP1A* and *SYNGAP1* in module 2 (Table 2, Supplementary Table S4).

**Table 2:** Likelihood ratio test (LRT) statistics for models of variable selective pressure among sites in the mammalian phylogeny (codon frequency: F3 × 4).

		M7-M8		M8a-M8		Sites (aa)^c^
		^−^ *2ΔlnL* ^*a*^	*p* value^b^	^−^ *2ΔlnL* ^*a*^	*p* value^b^	
M1	***MDM2***	33.357	1.142 × 10^−7^	4.683	3.047 × 10^−2^	I347, S348, T371, I372, I388, F420, V432
	***MECP2*** (***Reg1***)	11.692	5.782 × 10^−3^	7.593	5.860 × 10^−3^	—
	***MYC***	18.374	1.023 × 10^−4^	16.927	7.768 × 10^−5^	V185, I429
	***SMARCC2***	55.960	1.411 × 10^−12^	18.189	2.000 × 10^−5^	S1049
	***UIMC1***	147.783	1.623 × 10^−32^	87.994	6.566 × 10^−21^	S25, L152, T270, A424, F456, T604, S637, A648
M2	***GRIN2A***	82.719	2.181 × 10^−18^	58.105	2.485 × 10^−14^	V329, P1150
	***MAP1A***	189.749	1.252 × 10^−41^	147.643	5.678 × 10^−34^	P1065, D1080, A1115, H1259, T1277, Y1388, A1456, A1463, V1506, V1806, H2336, C2357
	***SYNGAP1*** (***Reg1***)	33.228	1.218 × 10^−7^	17.161	3.433 × 10^−5^	—

Models: M7 is a null model that assumes that 0 < ω < 1 is beta distributed among sites; M8 (positive selection model) is the same as M7 but also includes an extra category of sites with ω > 1. M8a is the same as M8, except that the 11^th^ category cannot allow positive selection, but only neutral evolution.

^a^2ΔlnL: twice the difference of the natural logs of the maximum likelihood of the models being compared.

^b^ *p* values are FDR corrected.

^c^Positions refer to the human sequence (see Supplementary Table 1, Supplementary material).

BEB^30^, FUBAR (Fast Unconstrained Bayesian AppRoximation)^38^, and REL (Random effects likelihood)^39^ were used to identify selected sites. Again, only sites detected by at least two different methods were considered as selection targets (Table 2).

Using this approach, an additional selected site (P1150) was detected in the GRIN2A CTD. Several positively selected sites were found in MDM2 and UIMC1. In MDM2, most selected sites are located in a C-terminal region encompassing two Zinc-finger domains; in UIMC1, sites tend to be scattered across the protein sequence (Fig. 3). Two of them (L152 and F456) are in a region necessary for the interaction with NR6A1^40^, a nuclear receptor with a role in neurogenesis^41^.

Among M2 genes, we identified 12 selected sites in *MAP1A*. This gene encodes a microtubule-associated protein predominantly expressed in neurons^42^. All selected sites are in the heavy-chain domain that cooperates with the light-chain for microtubule binding^43^. In particular, four selected sites are located in the second microtubule-binding domain (Fig. 3). Interestingly, one positively selected site was also detected in *SMARCC2*, which encodes a subunit of the chromatin remodeling complex mSWI/SNF that directly controls neurogenesis in the developing cerebral cortex by regulating its size and thickness^44^.

### Purifying and positive selection in humans and great apes

Taking advantage of the availability of genetic diversity data for humans and great apes, we combined analysis of intra-species polymorphism and between-species divergence to detect sites targeted by positive selection in the human, chimpanzee, and gorilla lineages. To this aim, we used the gammaMap method^45^, which categorizes population-scaled selection coefficients (γ) into 12 classes, ranging from strongly beneficial (γ = 100) to inviable (γ = −500), with γ equal to 0 indicating neutrality.

**Figure 4:**
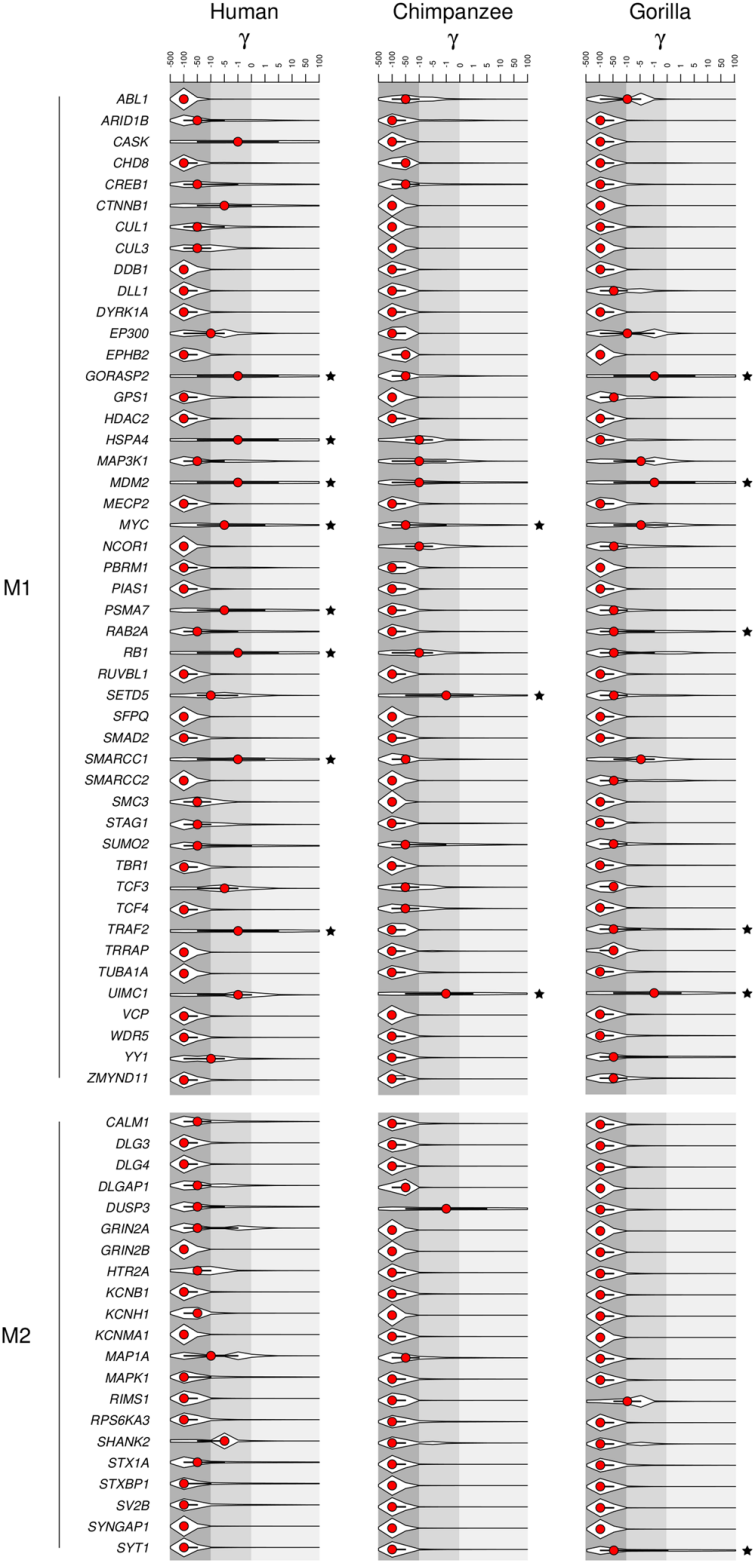
Population genetics-phylogenetics analysis in human, chimpanzee and gorilla lineages. Violin plot of selection coefficients for the the human, chimpanzee and gorilla lineages (median, red dot; interquartile range, black bar) for M1 and M2 genes. The gray shading denotes different degree of constraint based on selection coefficients. Black stars indicate genes with lineage-specific positively selected sites.

As expected, analysis of γ for M1 and M2 genes indicated a major role of purifying selection: most median values were lower than or equal to −50 (indicating that most amino acid replacements are deleterious) (Fig. 4). However, the degree of constraint was stronger in the gorilla and chimpanzee lineages compared to humans, especially for M1 genes (Fig. 4).

We also identified lineage-specific positively selected sites, defined as those with a cumulative probability higher than 0.75 of having γ ≥ 1^46^ (Table 3). Positively selected sites were detected in genes showing higher median γ values, suggesting that these genes were targeted by positive selection, and did not merely experience a relaxation of constraint or accumulated rare variants as a result of human population size growth^47^ (Fig. 4).

**Table 3:** Positively selected sites in the human, chimpanzee and gorilla lineages.

Gene	Lineage	Codon	Ancestral AA	Derived AA	Pr^a^
*GORASP2*	Human	257	Ala	Thr	0.901
	Gorilla	247	Pro	Ser	0.988
*HSPA4*	Human	778	Ile	Thr	0.982
*MDM2*	Human	283	Arg	Gln	0.960
	Human	432	Met	Val	0.961
	Gorilla	413	Ser	Cys	0.948
*MYC*	Human	369	Asp	Val	0.874
	Chimp	224	Ala	Pro	0.872
*PSMA7*	Human	216	Pro	Ser	0.911
*RAB2A*	Gorilla	197	Thr	Ser	0.752
*RB1*	Human	233	Val	Met	0.947
*SETD5*	Chimp	421	Thr	Ala	0.759
	Chimp	563	Pro	Ala	0.760
*SMARCC1*	Human	117	Thr	Ala	0.850
	Human	437	Pro	Leu	0.851
*SYT1*	Gorilla	420	Val	Ile	0.902
*TRAF2*	Human	237	Ala	Val	0.963
	Human	258	Ser	Leu	0.963
	Human	373	Thr	Ile	0.935
	Gorilla	221	Ile	Val	0.781
*UIMC1*	Chimp	429	Arg	Gly	0.958
	Chimp	439	Ala	Thr	0.960
	Chimp	511	Arg	His	0.911
	Chimp	597	Ser	Gly	0.974
	Chimp	601	Cys	Phe	0.974
	Gorilla	16	Asn	Ile	0.913
	Gorilla	62	Thr	Ala	0.894
	Gorilla	502	Gln	His	0.764
	Gorilla	581	Ser	Cys	0.775

^a^Posterior probability of γ ≥ 1 as detected by gammaMap.

### Purifying selection in human populations

**Figure 5:**
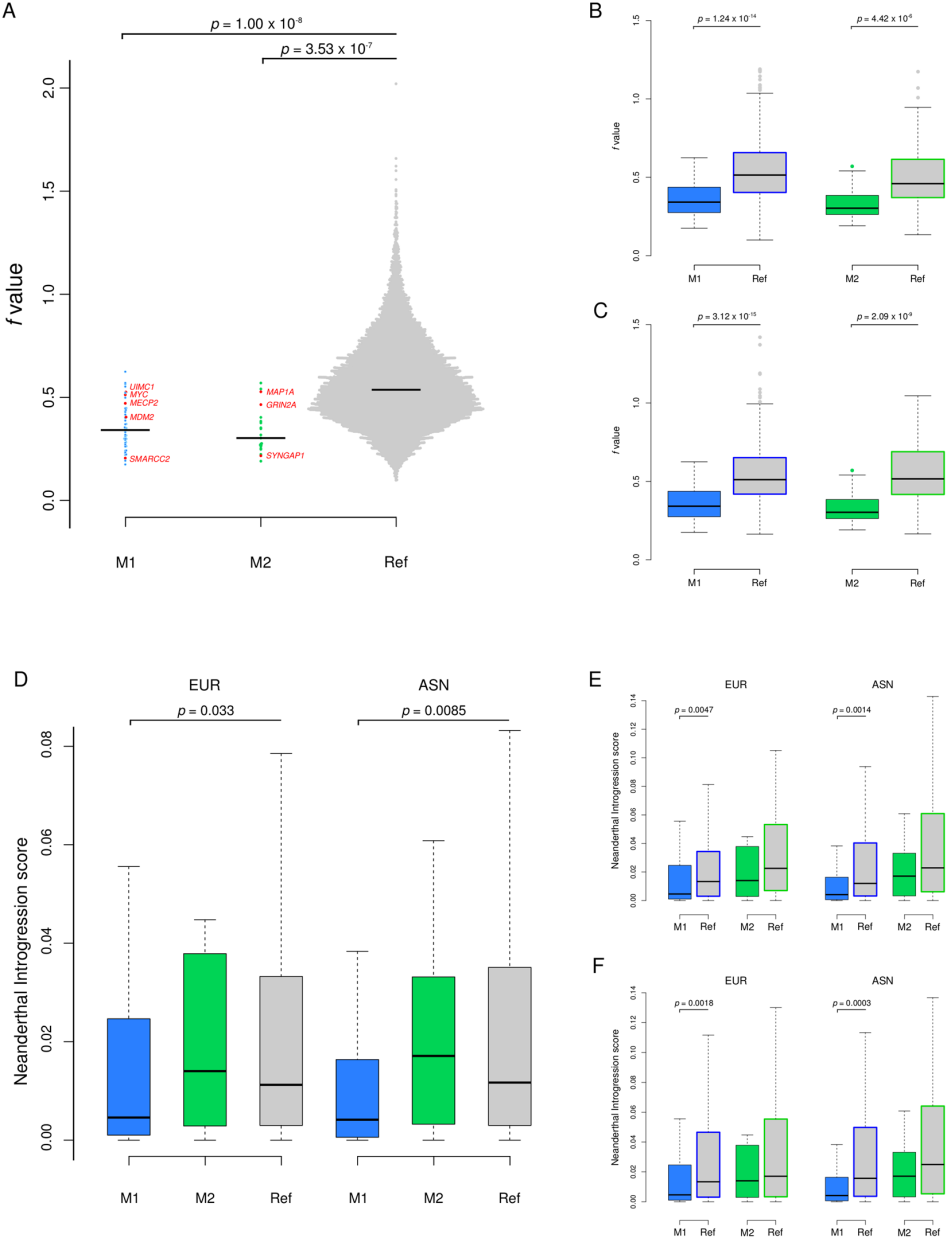
Degree of selective constraints and Neanderthal ancestry of M1 and M2 genes. (**A**) Swarm plot representation of *f* values for M1 (blue), M2 (green), and all other human RefSeq genes (Supplementary Table S5). Tukey’s post-hoc test *p* values are reported. Genes showing evidence of positive selection in the mammalian phylogeny are in red. (**B**) Boxplot representation of *f* values for M1 and M2 genes compared to gene sets matched for GC content and length (M1 reference set (blue border) = 1344 genes, M2 reference set (green border) = 84 genes) or (**C**) GERP score (M1 reference set (blue border) = 649 genes, M2 reference set (green border) = 126 genes). Student’s *t* test *p* values are reported. (**D**) Comparison of the average introgression score in European (EUR) and East Asian (ASN) population for M1, M2 and all RefSeq genes. Nemenyi’s post-hoc test *p* values are reported. (**E**) Comparison of average introgression scores between M1/M2 genes and gene sets matched by GC content and length or (**F**) GERP scores. Wilcoxon Rank-Sum test *p* values are reported.

Given the major effect of purifying selection in driving the evolution of M1 and M2 genes, we next compared their level of constraint to that imposed on other human genes. To this purpose, we used SnIPRE, which contrasts polymorphism and divergence data at nonsynonymous and synonymous sites, to calculate the constraint parameter *f*. *f* represents the proportion of mutations that are non-lethal. Thus, the lower *f* is for a given gene, the stronger its level of constraint^48^. *f* values were calculated for genes in modules 1 and 2, as well as for all human RefSeq autosomal coding genes (see Materials and Methods). The distribution of *f* values was significantly different in the three groups (one-way ANOVA, F = 39.5, *p* = 2 × 10^−16^), with both M1 and M2 genes showing significantly lower average *f* compared to all other human genes (Tukey’s test, *p* = 1.00 × 10^−8^ and *p* = 3.53 × 10^−7^, respectively) (Fig. 5A, Supplementary Table S5). Because the degree of constraint may depend on gene features unrelated to function (e.g., GC content)^49, 50^, we compared genes in the M1 and M2 modules to gene subsets matched for GC content and length (see methods for matching procedures). M1 and M2 genes showed significantly lower *f* values when compared to the respective matched gene sets (Fig. 5B). Finally, we wished to verify whether M1 and M2 genes differ in the level of coding sequence constraint compared to genes that display similar evolutionary rates over their entire length (coding and non-coding) and across a longer time-frame. We thus used GERP (Genomic Evolutionary Rate Profiling) scores to obtain gene sets matched to genes in the M1 and M2 modules. Again M1 and M2 genes displayed lower *f* values (Fig. 5C).

### Neanderthal introgression and modern human-specific alleles

Admixture with extinct hominins (Neanderthals and Denisovans) resulted in the introgression of archaic alleles into the human gene pool^51–53^. Recent data indicated that genomic regions experiencing strong levels of background selection are depleted in Neanderthal ancestry^54^. Moreover, regions depleted of both Neanderthal and Denisova ancestry are enriched for genes expressed in specific brain regions (e.g. the ventral frontal cortex-ventrolateral prefrontal cortex in infants and the striatum in adulthood)^55^. We thus used a Neanderthal introgression map to estimate the average introgression scores for M1 and M2 genes. These scores were compared to those calculated for all human coding genes to determine whether M1 and M2 genes experienced unusual levels of introgression. Significant differences among the three groups (M1, M2, and all other genes) were observed for both Europeans and Asians (Kruskall-Wallis test, *p* = 0.032 and = 0.0085, respectively) (Fig. 5D, Supplementary Table S5). Specifically, M1 genes showed significantly lower levels of introgression compared to all coding human genes in both populations (Nemenyi post-hoc test, *p* = 0.033 and *p* = 0.0085 for Europeans and Asians, respectively) (Fig. 5D). The same results were obtained when M1 genes were compared to gene sets matched in GC content and length or GERP scores (Fig. 5E and F).

**Figure 6:**
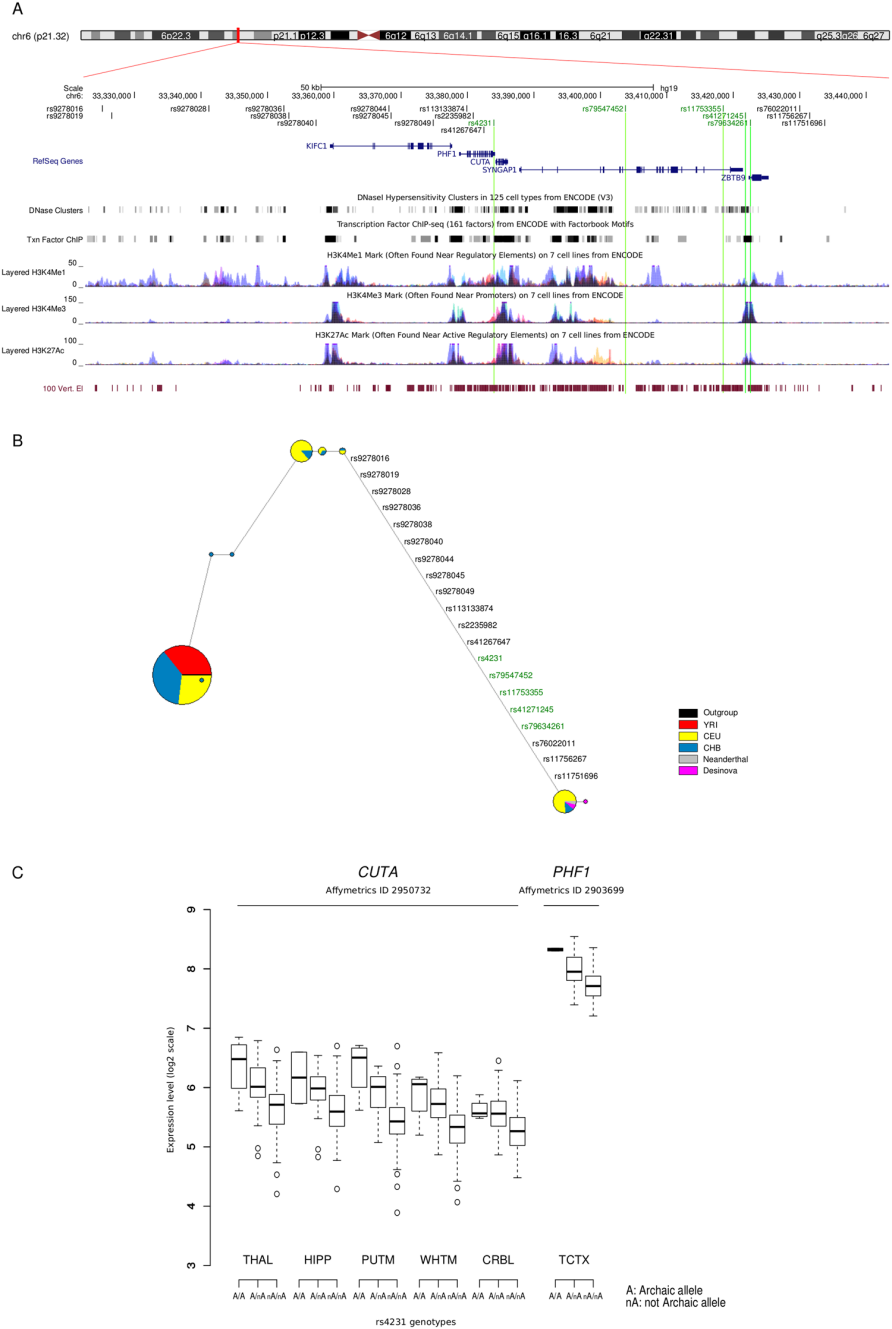
Neanderthal introgression at the *SYNGAP1* locus. (**A**) The genomic region containing *SYNGAP1* is shown within the UCSC Genome Browser view. The 20 archaic variants defining the introgressed Neanderthal haplotype are reported. Relevant annotation tracks are also shown. SNPs reported as eQTLs in BRAINEAC are in green. (**B**) Haplotype analysis reconstructed through a median-joining network of *SYNGAP1* introgressed SNPs. Each node represents a different haplotype, with the size of the circle proportional to frequency. IDs for SNPs defining the haplotype shared by Neanderthals, Denisovans, and a small fraction of non African modern humans are listed on the branch. (**C**) Box plot representation of *CUTA* and *PHF1* genes expression levels stratified by genotype status at rs4231. Data derive from the BRAINEAC data collection (THAL, thalamus; HIPP, hippocampus; PUTM, putamen; WHMT, white matter; CRBL, cerebellum; TCTX, temporal cortex).

However, this finding does not imply that introgression did not occur at these genes. In fact, 8 genes in either M1 or M2 had an introgression score higher that the 95^th^ percentile value calculated on the distribution of all human coding genes (Supplementary Table S5). These genes were further analyzed by identifying regions with a high introgression score and by searching, within these regions, for archaic variants (i.e. homozygous positions in the Neanderthal sequence where the archaic allele is present in populations of non-African ancestry only). Among archaic SNPs, we searched for those with likely functional effects by identifying brain eQTLs (via the BRAINEAC database). One ~108 kb haplotype defined by 20 archaic SNPs in full linkage disequilibrium was found to span *SYNGAP1* and nearby genes (Fig. 6). Network analysis of these variants identified three major haplotypes one of which is shared by Neanderthals, Denisovans, and by a small fraction of non African modern human chromosomes (Fig. 6). Five variants defining the introgressed haplotype are reported as eQTLs in BRAINEAC. However, these SNPs do not represent eQTLs for *SYNGAP1*, but rather modulate the expression of two nearby genes: *CUTA* (CutA divalent cation tolerance homolog protein) and *PHF1* (PHD finger protein 1). Specifically, for both genes the archaic SNPs are associated with higher expression level in different brain areas (Fig. 6).

**Figure 7:**
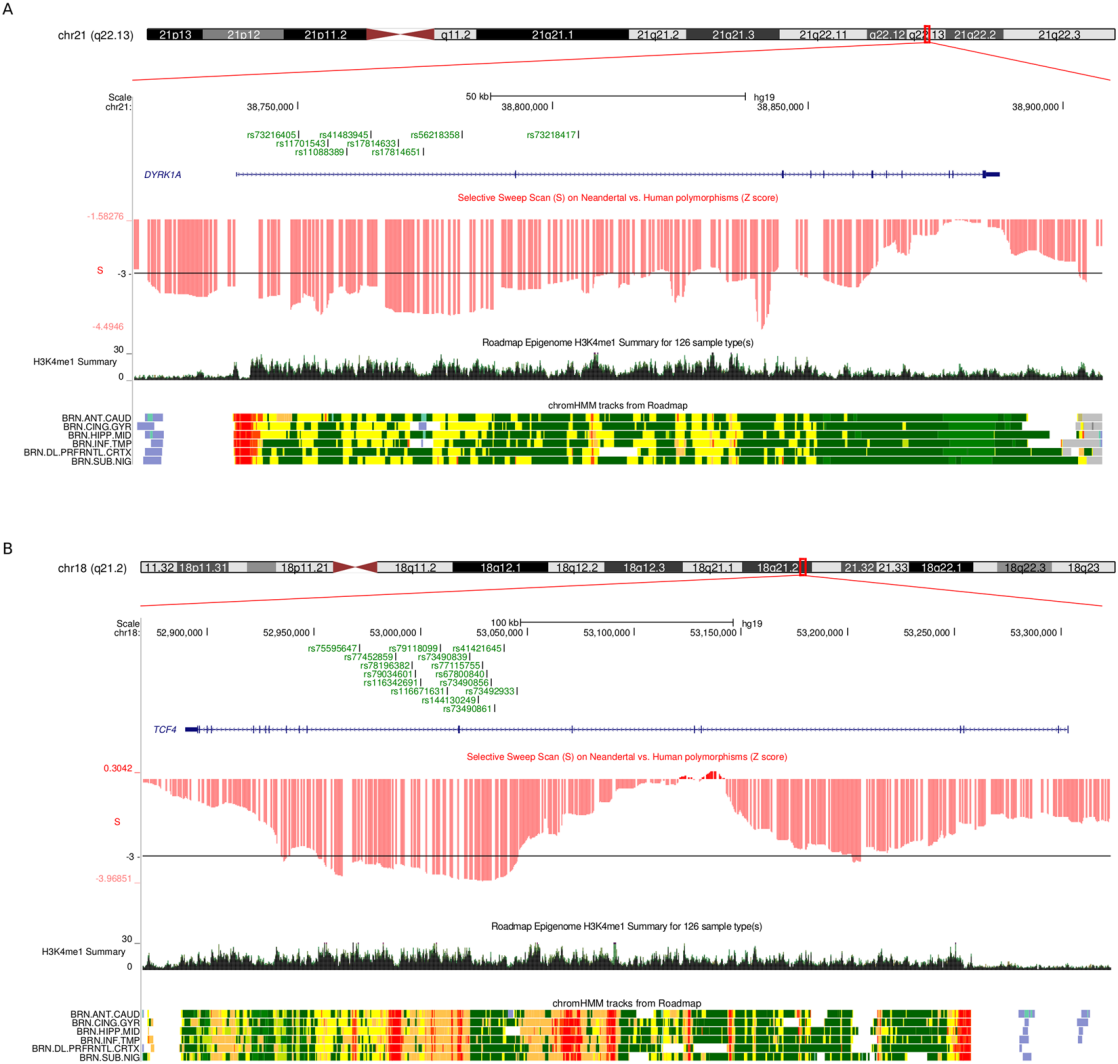
*DYRK1A* and *TCF4* modern human alleles. Modern-human-specific SNPs and their location in *DYRK1A* (**A**) and *TCF4* (**B**) are shown within the UCSC Genome Browser view. S scores are shown in red. The horizontal black line represents the 5^th^ percentile of S score. Relevant annotation tracks from the *Roadmap Epigenomics Data Complete Collection at Wash U Viz Hub* are also shown, filtered for brain tissues only.

The availability of archaic hominin sequence data also allows the identification of modern human alleles – i.e., alleles that were absent in Neanderthals and Denisovans but display high frequency in modern human populations. We used a catalog of modern-human-specific sites to search for variant located in M1 or M2 genes^56^. Several modern alleles were identified and these were filtered by requiring that: (i) both the Altai Neanderthal and the high-coverage Denisova sequence were homozygous for the ancestral allele, and (ii) the variants were either eQTLs in brain or mapped to putative regulatory regions for brain expression (as assessed by open chromatin, histone modifications or DNAse hypersensitivity, see methods). We next assessed whether variants passing these criteria (n = 138) were located in regions that experienced positive selection in early modern human populations. To this aim, we exploited the selection scan score (S) developed by Green and coworkers^51^. S is negative in regions where Neanderthals carry fewer derived alleles than expected based on the allelic configuration in modern populations, a scenario consistent with selection in early modern humans. Specifically, we called selected regions in M1 and M2 genes as windows of at least 25kb where all SNPs have an S score lower than the 5^th^ percentile (calculated on the genome-wide distribution of S scores, see methods). A few modern human-specific variants in *YY1*, *STAG1*, and *PIAS1* occurred in putative selected regions (Supplementary Table S6 and Supplementary Fig. S1). However, the strongest signals were evident for *DYRK1A* and *TCF4*, which showed several modern human variants in long regions of low S (Fig. 7). Indeed, *DYRK1A* had been identified as a top candidate for early human selective events by Green and coworkers^51^. As expected, given the selection criteria of high frequency in modern human populations, the derived alleles of *DYRK1A* and *TCF4* variants are fixed in populations of Asian ancestry, while they display very high frequency in Europeans and Africans (Supplementary Table S6).

## Discussion

We took advantage of genetic diversity data for human populations, archaic hominins and great apes, as well as of genomic information for vertebrates to provide insight into the evolution of two gene modules involved in neurodevelopmental disorders. We focused in particular on 68 genes which, despite not being an exhaustive catalog of genetic risk loci for ASD and ID, were previously shown to represent functional modules enriched of *de novo* mutations in probands^29^. Whereas module 2 is rather homogeneous in terms of protein function, M1 comprises diverse functional categories, including genes involved in the ubiquitin pathway and in cancer (see below), in line with previous observations^57, 58^.

We first assessed whether M1 and M2 genes evolved under different selective pressure on the branches leading to Mammalia and to Sauropsida. This was achieved by using branch-site tests, which were shown to be robust to the large evolutionary distances of the vertebrate phylogeny^59^. Although lineages showing high cognitive skills are reported both among mammalian and bird species, mammals are generally believed to have acquired higher prosocial skills compared to non-mammalian vertebrates^60, 61^. For instance, prosocial behaviors have been documented in primates and rodents^60, 61^. Conversely, experiments in crows, birds that display high cognitive abilities^10^, failed to reveal other-regarding preferences and reciprocity^11, 12^.

We did not observe a substantial difference in selective pressure in Mammalia compared to Sauropsida, and in most cases we detected evidence of episodic positive selection for the same genes in both lineages, suggesting that coding variants in these genes do not represent major drivers of social skill differences among Vertebrata. However, sites that experienced episodic positive selection in either the mammalian or the sauropsidan branch were detected in several genes with clear involvement in ASD or ID. In this respect it is worth noting that branch-site tests have low false positive rates but also limited power to detect specific sites targeted by selection^30^. Moreover, we applied a conservative criterion by requiring that selected sites were identified by at least two methods. These factors are likely to have resulted in an under-estimation in the number of positively selected sites, and in some instances no site was detected despite a significant LRT. We thus extended evolutionary analysis to a larger mammalian phylogeny and we used a population genetics-phylogenetic approach to more specifically investigate the strength of selection acting on humans and great apes.

Based on the number of detected sites and on coding sequence length, the genes showing the strongest signals of positive selection in Mammalia and in Hominindea were *MDM2* and *UIMC1*. Both genes are highly expressed in the testis (http://www.gtexportal.org/) and have been implicated in cancer^62, 63^. *MDM2* encodes a nuclear-localized E3 ubiquitin ligase that mediates the degradation of p53 and RB1^62^ in a proteasome-dependent and ubiquitin-independent manner^64^. The protein product of *UIMC1* (often referred to as RAP80) is a nuclear protein involved in the multivalent recognition of polyubiquitin chains (UIMs) that recruits BRCA1 and other proteins to DNA damage sites^65^. Previous works reported that genes involved in cancer and apoptosis, including *BRCA1* and *BRCA2*, are common targets of positive selection^66, 67^. In line with these data, we also found selected sites in *MYC*, *RB1*, and *TRAF2*, genes which play a role in cancer and/or apoptosis (Fig. 4, Table 3). An interesting possibility to explain these findings is selfish spermatogonial selection: variants that increase the rate of cell division or decrease the probability of apoptosis in a given germline cell are favored, irrespective of the fitness effect on the embryo^68^. Indeed, male germ-line-selective advantage has previously been described for mutations in other genes involved in cancer (e.g. *FGFR2*, *FGFR3*, *RET*, *PTPN11*) that cause congenital disorders with paternal age effect^69–77^. In this respect, it is worth noting that missense mutations in probands with ASD were described in MDM2 and UIMC1 regions where positively selected sites are also located (Fig. 3). Intriguingly, the MDM2 region where the selected sites and the ASD mutation map is necessary for USP2a binding, which results in MDM2 stabilization and p53 degradation^78^. The *MDM2* mutation detected in the ASD proband was of paternal origin (no information is available for the *UIMC1* change)^79^. Indeed, recent analyses reported a strong paternal bias in *de novo* ASD point mutations and a correlation between mutation number and paternal age^79^. Given that a substantial overlap exists in risk genes for autism and for cancer^58^, it will be important to assess whether a subset of mutations associated with ASD derive from selfish spermatogonial selection.

*MDM2* and *UIMC1* were identified as top risk genes for ASD, although the contribution of specific variants remains to be validated. Based on functional data, *MDM2* is a very promising candidate. In mouse neurons, MDM2 ubiquitinates DLG4 (also known as PSD-95) and participates with other ASD-associated genes to the process of experience-dependent synapse elimination^80^. PSD-95 is a membrane-associated guanylate kinase (MAGUK) acting as scaffold for junctional surface complexes and actin cytoskeleton, thus contributing to the organization of the postsynaptic density (PSD)^81^. Whereas the evolutionary history of DLG4 seems to be dominated by purifying selection, three of its direct interactors (*GRIN2A*, *SYNGAP1*, and *MAP1A*, in addition to *MDM2*) showed evidence of positive selection on the mammalian branch. Mutations in *GRIN2A* have been associated with a variety of neurological disorders^82^. NMDA receptors are both ligand-gated and voltage-dependent, and play a fundamental role in brain development and function. Interestingly, three of the four positively selected sites detected in *GRIN2A* are in the intracellular CTD domain, which contains the terminal conserved -ESDV- sequence required for the interaction whit DLG4^83^. This domain primarily experienced diversification during the two rounds of gene duplication that led to the generation of four GluN2A-D paralogs in Vertebrata^84^. CTD diversification led to the development of subunit-specific functions in the regulation of vertebrate behavior, depending on the differential modulation of synaptic signaling. In particular, the CTDs of GRIN2A and GRIN2B (GluN2B) differentially regulate behavioral phenotypes in mice (e.g. impulsivity and anxiety)^85^. This difference is partially mediated by the differential binding to intracellular signaling proteins^85^. Reverse-genetics experiments will be required to evaluate whether variation at the positively selected sites modulate phenotype traits in mammals or NMDAR biochemical properties.

With respect to *SYNGAP1* and *MAP1A*, both encoding abundant proteins in the PSD, very different numbers of positively selected sites were observed. Several sites were detected in *MAP1A*, whereas only few are located in *SYNGAP1*. This difference is likely to reflect both the strength of positive selection acting on these genes and the relevance of functional/structural constraint in limiting the space accessible for amino acid substitutions. At least in Homininae and in human populations, *SYNGAP1* and *MAP1A* display different levels of constraint. *MAP1A* is relatively tolerant to amino acid substitutions compared to other genes in the M1 and M2 modules (Fig. 5). This observation does not imply that the gene is dispensable, and loss of *MAP1A* function causes neurodegeneration in mice^86^, but some changes that do not abolish protein function can likely be tolerated. In this respect, it is worth noting that *de novo* mutations in *MAP1A* have not been unequivocally associated to ASD or ID, but a population genetics study indicated that the gene is significantly enriched in rare missense variants when ADS and SCZ subjects are compared to controls^87^. This observation, together with our data, suggests that mildly deleterious variants in *MAP1A* segregate at low frequency and contribute to the genetic susceptibility to ASD/SCZ. *SYNGAP1*, on the contrary, appears to be strongly constrained and only two selected sites were identified. Interestingly, one of them is located in the C2 domain. *De novo* mutations in *SYNGAP1* are thought to represent a relatively common cause of ID with epilepsy, and most detected changes in affected subjects are loss-of-function mutations^33^. Five missense variants have been reported to date as pathogenic and two of them are located in the C2 domain, indicating that amino acid substitutions in this region potentially modulate cognitive phenotypes^33^.

Whereas the level of constraint was similarly high at M1 and M2 genes, only genes in the M1 module were found to display significantly lower Neanderthal introgession scores compared to the reference gene set. The reason(s) why genomic regions experiencing background selection tend to be depleted in Neanderthal ancestry is a still matter of debate^54, 88^. Both epistatic reproductive incompatibilities between humans and Neanderthals and increased mutation load due to reduced fitness in Neanderthals have been proposed as possible explanations^54, 88^. Disentangling these alternatives is beyond the scope of our work. As for the reason why the M2 module did not show reduced Neanderthal introgression, we note that it comprises few genes, and most of these tend to display low introgression scores. The high average score of the M2 module is largely due to few outliers in CEU (*GRIN2B*, *SYNGAP1*, and *KCNH1*) and ASN (*MAPK1*) (Supplementary Table S5).

All the archaic SNPs we identified in genes with high introgression scores were located in non-coding regions. Because eQTLs for cerebellum and temporal cortex were found to be over-represented among introgressed SNPs^89^, we checked our archaic variants against the BRAINEAC database. BRAINEAC provides information on eQTLs from 12 brain regions obtained from 134 neurologically healthy individuals of European descent^90^. Results showed the presence of a *SYNGAP1* introgressed haplotype shared by Neanderthals, Denisovans, and by a small fraction of non African modern humans. Five SNPs in the haplotype are reported as eQTLs for two nearby genes, *CUTA* and *PHF1*. Although these genes are involved in different processes, both regulate the signaling of molecules acting as neurotransmitters, namely acetylcholyne and γ-aminobarbituric acid (GABA). CUTA affects the folding, oligomerization, and secretion of acetylcholynesterase^91^, whereas the PHF1b isoform promotes the transcription of *GABRB1*, which encodes GABA type A receptor (GABA_A_R); this results in the regulation of GABA-mediated neurotransmission in the central nervous system, in particular in neocortical and hippocampal neurons^92^. CUTA also modulates the generation of β-amyloid peptides (Aβ), major components of senile plaques typical of Alzheimer’s disease^93^. Specifically, the longest isoform of CUTA interacts with BACE1, a β-secretase involved in the generation of Aβ peptides, reducing secretion of neurotoxic molecules. We found that the introgressed allele at rs4231 (C) is associated with an increased expression of CUTA in many brain tissues, suggesting a protective effect against neurotoxic β-amyloid plaque generation. It is tempting to speculate that, as both cholinergic and GABAergic signaling are finely regulated, changes in the expression levels of *CUTA* and *PHF1* would result in alterations of synaptic plasticity^94, 95^. These data are in line with recent observations whereby introgressed alleles are often associated with neurological disorders^89^ and changes in methylation patterns between modern humans and archaic hominins are common at genes involved in neurological and psychiatric diseases^96^. However, it remains to be evaluated whether the introgressed variants we describe entail a phenotypic effect.

Likewise, the functional significance of the modern-human-specific variants we detected in *DYRK1A* and *TCF4* is presently unknown. It is also worth mentioning that modern-human-specific alleles are defined on the basis of two archaic genomes only. Thus, these alleles may have been present in Neanderthals and/or Denisovans, possibly at low frequency, and be unsampled in these two individuals. Alternatively, derived alleles at these sites may have existed before the split of humans from archaic hominins and be lost to drift in Neanderthals and Denisovans. Nonetheless, the fact that several putative modern alleles in *DYRK1A* and *TCF4* are located in regions showing signatures of selection in early humans supports the view that some selective pressure drove their frequency increase in modern populations.

Mutations in *DYRK1A* and *TCF4* cause syndromic diseases presenting with microcephaly and intellectual disability^97–105^. For both genes haploinsufficiency is often associated with disease, indicating that adequate expression levels of these proteins are essential for neurodevelopment^98, 101, 106^. Moreover, in the case of *DYRK1A*, over-expression can also be deleterious: the gene is located in the Down syndrome (DS) critical region and its copy number alteration may be the underlying mechanism for abnormal brain development in DS^107^. Thus, fine-tuning of *DYRK1A* expression is central to normal brain development. The putative modern alleles we detected in these two genes are located within regions that display chromatin features typical of transcriptional enhancers in several brain areas, strongly suggesting a regulatory role on gene expression. These SNPs thus represent candidates for association with neurodevelopmental disorders, and await experimental validation in future studies (eg., by approaches that exploit genetically-manipulated induced pluripotent stem cells).

## Methods

### Datasets and Databases

We analysed the evolutionary history of 68 genes identified using MAGI, a computational method that simultaneously integrates data of protein-protein interaction and co-expression network to identified sets of genes defined “disease modules” enriched in *de novo* mutation in cases compared to controls^29^.

In particular, we focused on the two gene sets denoted by Hormozdiari^29^ as *Best Modules* (47 genes in M1 and 21 in M2) (Fig. 1, Supplementary Table S1).

Gene coding sequences were retrieved from the Ensembl (http://www.ensembl.org/index.html) and the National Center for Biotechnology Information (NCBI, http://www.ncbi.nlm.nih.gov) databases.

1000 Genomes Phase 1 data for population genetics analysis were retrieved from the dedicated website (http://www.1000genomes.org/). The marginal probabilities of Neanderthal ancestry for Europeans and Asians were retrieved from the Datasets-Neanderthal Introgression (http://genetics.med.harvard.edu/reichlab/Reich_Lab/Datasets_-_Neandertal_Introgression.htmlgenetics.med)^54^, whereas the list of “modern-human-specific sites” were obtained from Prufer and colleagues^56^.

Brain eQTLs data were retrieved from the the Brain eQTL Almanac (BRAINEAC) database (www.braineac.org/)^90^.

S scores for all SNPs in the genome were retrieved from the UCSC genome browser (table name: Selective Sweep Scan (S))^51^.

### Evolutionary analysis in vertebrates

We identified orthologous coding sequences in the genome of 33 species selected to be representative for the Vertebrata subphylum and to include a similar number of mammalian and sauropsidan species (Fig. 2, Supplementary Table S2).

Orthology was assessed using the EnsemblCompara GeneTrees database^108^ and only 1-to-1 orthologs were included. Because this database does not include some Sauropsida species (*Falco peregrinus*, *Zonotrichia albicollis*, *Melopsittaus undulatus*, *Columba Livia* and *Alligator mississippiensis*), we performed BLAST searches of the human coding sequences against the genome of these species using the NCBI BLAST utility. Hits that were not consistent with the presence of a single ortholog were removed. *SUMO2* and *YY1* (M1) were not included in this analysis due to the impossibility to reach a substantial number of orthologs from other species.

Alignments were performed using the RevTrans 2.0 utility^109^ and manually edited to remove uncertainties in proximity of small gaps.

Substitution saturation was checked using Xia’s index implemented in DAMBE^110^. This test compares a entropy-based index of saturation (I_ss_) with a critical value (I_ss.c_). If I_ss_ is significantly lower than I_ss.c_, sequences have not experienced substitution saturation. No evidence of significant saturation was obtained for any alignment.

All alignments were screened for the presence of recombination breakpoints using GARD^111^. GARD evaluates the statistical significance of putative breakpoints through Kishino-Hasegawa (HK) tests. A breakpoint was considered significant if its *p* values were lower that 0.01.

The branch-site likelihood ratio tests (models MA and MA1) implemented in the PAML suite^37^ were used to test the mammalian and sauropsidan branches.

The total tree length for the gene alignments ranged from 3.17 to 18.15; these values are within a good accuracy range for codeml sites models^112^. We used two different codon frequencies model: the F3 × 4 model (codon frequencies estimated from the nucleotide frequencies in the data at each codon site) and the F61 model (frequencies of each of the 61 non-stop codons estimated from the data)^37^. An FDR correction was applied to account for multiple hypothesis testing, as suggested^31^. Positively selected sites were identified using two different methods: the Bayes Empirical Bayes (BEB) analysis from MA (with a cutoff of 0.90) and the Mixed Effects Model of Evolution (MEME) (with the default cutoff of 0.1)^34^.

GARD and MEME analyses were performed either through the DataMonkey server^113^ (http://www.datamonkey.org) or run locally^114^.

### Evolutionary analysis in the mammalian phylogeny

Coding sequences were retrieved for at least 60 mammalian species, including Metatheria and Eutheria (Fig. 2, Supplementary Table S2). Sequences were checked for orthology, aligned, and screened for recombination as described in the previous section.

To detect positive selection, we used the *codeml* NSsite models from PAML. Selection was declared if both neutral models (M8a, M7) were rejected in favor of the positive selection model (M8) using the F3 × 4 and F61 codon frequency models.

When the likelihood ratio test indicated the action of positive selection, we applied three different methods to identified individual selected sites: BEB analysis (from M8 with a cutoff of 0.90)^112^, the Random effects likelihood (REL, with a cutoff of 50), and the Fast Unconstrained Bayesian AppRoximation (FUBAR, with a cutoff of 0.90). To limit false positives, we considered a site as positively selected if it was detected by at least two different methods.

### Population genetics-phylogenetics analysis in the human, chimpanzee, and gorilla lineages

Human data derive from the 1000 Genomes Phase 1 Project database for European (CEU), Yoruba (YRI), and Chinese (CHB). For chimpanzees and gorillas, we used SNP information from 25 and 27 individuals, respectively^115^.

Ancestral sequences were reconstructed by parsimony from the human, chimpanzee, orangutan, and macaque sequences.

Analyses were performed with gammaMap, that evaluates intra-specific variation and inter-specific diversity to estimate, along coding regions, the distribution of population-scaled selection coefficients (γ). In this framework, *γ* is defined as  2PNes, where P is the ploidy, Ne is effective population size, and s is the fitness advantage of any amino acid-replacing derived allele.

In the analysis, we assumed θ (neutral mutation rate per site), k (transitions/transversions ratio), and T (branch length) to vary among genes following log-normal distributions, whereas p (probability of adjacent codons to share the same selection coefficient) following a log-uniform distribution. For each gene we set the neutral frequencies of non-STOP codons (1/61). For selection coefficients we considered a uniform Dirichlet distribution with the same prior weight for each selection class. For each gene we run 100,000 iterations with thinning interval of 10 iterations.

### Purifying selection in humans

The strength of purifying selection was estimated using SnIPRE^48^, a tool that relies on the comparison of polymorphism and divergence data from synonymous and non-synonymous sites within genes. SnIPRE uses a generalized linear mixed model to represent the genome-wide variability among categories of mutations and to estimate its functional consequence. We estimated the degree of selective constraints at each gene using the *f* parameter, which is the proportion of non-synonymous mutation that are not deleterious.

The *f* parameter was estimated for each gene of the M1 and M2 modules and for all RefSeq autosomal coding human genes used as reference (Supplementary Table S5).

To evaluate divergence within genes, we used the liftOver tool to convert human GRCh37/hg19 genome coordinates to *Pan troglodytes* (CGSC 2.1.3/PanTro3) coordinates; we selected only genes that mapped on chimpanzee genome (n = 14805).

Specific reference gene sets were selected for both the M1 and M2 modules. These sets were obtained by controlling for base composition and gene length or gene conservation. We used a treshold of ±10% for each feature and a matching procedure similar to that reported by Enard and colleagues^116^. Thus, for each M1 and M2 gene, we searched for all matching genes whose GC content and length or GERP score differed less or more than 10% from those of the M1/M2 gene. GERP scores were obtained from UCSC genome browser (table name: GERP Scores for Mammalian Alignments)^117^.

### Neanderthal introgression and haplotype analysis

To investigated the introgression from archaic hominins, we used the probabilities of Neanderthal ancestry calculated for each SNP of the 1000 Genomes Project dataset^54^. We used the inferred Neanderthal ancestry at each allele in European (CEU, GBR, FIN, IBS and TSI) and Asian (CHB, CHS, and JPT) populations^54^. The introgression summary score was calculated for each gene by averaging the marginal probabilities of Neanderthal ancestry for all SNPs of the gene.

We thus estimated the average introgression scores for M1 and M2 genes, as well as for all RefSeq coding human genes. We analyzed in detail M1 and M2 genes having an average introgression score higher that the 95^th^ percentile value based on the distribution of all genes. Introgressed regions were defined based on the presence of archaic variants (i.e. homozygous positions in the Neanderthal sequence where the archaic allele is only present in populations of non-African ancestry) with an high introgression score (i.e. higher than 95^th^ percentile). The introgressed regions were then analyzed for the presence of brain eQTLs (via the BRAINEAC database) and median-joining networks^118^ were constructed to infer haplotype genealogy. For Network analyses we used CEU, YRI, and CHB SNPs with genotype information from an Altai Neanderthal and a Denisovan individuals^52, 56^.

### Modern human alleles and selection in early modern humans

A list of modern human-specific sites - i.e. positions where the Denisova or Altai Neanderthal sequences display the ancestral allele, whereas most modern humans carry the derived allele - were retrieved from a previous study^56^. We filtered these variants by requiring that both the Altai Neanderthal and the Denisova sequences were homozygous for the ancestral allele and the variants were either eQTLs in brain or mapped to putative regulatory regions for brain expression. For this purpose, we used information from the HaploReg database^119^ to scan for variants in regulatory regions as assessed by open chromatin, histone modifications, or DNAse hypersensitivity.

To assess whether a moder human-specific SNPs were located in genomic regions that experienced positive selection in early modern humans, we exploited the selection scan score (S) developed by Green *et al*.^51^. A negative S score identifies a region where Neanderthals carry fewer derived alleles than expected based on the allelic status in modern populations. S scores were retrieved for all SNPs in the genome and the genome-wide distribution of S was calculated. We then searched for modern-human-specific variants in M1/M2 genes that were located in a region of at least 25 Kb where all SNPs have an S score lower than the 5^th^ percentile in the genome-wide distribution.

## Electronic supplementary material

Supplementary Information
