# Supplementary material for: Distinct selective forces and Neanderthal introgression shaped genetic diversity at genes involved in neurodevelopmental disorders
Source: Sci Rep. 2017 Jul 21;7:6116. doi: 10.1038/s41598-017-06440-4 (PMC5522412; doi:10.1038/s41598-017-06440-4)

Distinct selective forces and Neanderthal introgression shaped genetic diversity at genes involved in neurodevelopmental disorders

Alessandra Mozzi[1], Diego Forni[1], Rachele Cagliani[1], Uberto Pozzoli[1], Mario Clerici[2,3], and Manuela Sironi[1]

[1] Bioinformatics, Scientific Institute IRCCS E. MEDEA, 23842 Bosisio Parini, Italy.

[2] Department of Physiopathology and Transplantation, University of Milan, 20090 Milan, Italy.

[3] Don C. Gnocchi Foundation ONLUS, IRCCS, 20100 Milan, Italy.

Corresponding author: Alessandra Mozzi, PhD, Bioinformatics - Scientific Institute IRCCS E. MEDEA, 23842 Bosisio Parini, Italy. Tel: +39-031877826; Fax:+39-031877499; e-mail: alessandra.mozzi@bp.lnf.it

Supplementary Information

Captions:

Supplementary Table S1. List of M1 and M2 genes
Supplementary Table S2. List of Species
Supplementary Table S3. Likelihood ratio test (LRT) statistics for models of variable selective among Vertebrata branches (codon frequency:F61).
Supplementary Table S4. Likelihood ratio test (LRT) statistics for models of variable selective pressure among sites in mammalian phylogeny (codon frequency: F61).
Supplementary Table S5. *f*-values and Introgression scores.
Supplementary Table S6. Selected SNPs in early modern human populations.
Supplementary Figure S1. *PIAS1*, *STAG1*, and *YY1* modern human alleles

Supplementary Table S1. List of M1 and M2 genes.

Genes	Transcript ID	Protein Name	chr	start	end
M1					
ABL1	NM_007313	Tyrosine-protein kinase ABL1	chr9	133589267	133763062
ARID1B	NM_020732	AT-rich interactive domain-containing protein 1B	chr6	157099063	157531913
CASK	NM_003688	Calcium/calmodulin-dependent serine protein kinase	chrX	41374188	41782287
CHD8	NM_001170629	Chromodomain-helicase-DNA-binding protein 8	chr14	21853352	21899867
CREB1	NM_134442	Cyclic AMP-responsive element-binding protein 1	chr2	208394615	208470284
CTNNB1	NM_001904	Catenin beta-1	chr3	41240941	41281939
CUL1	NM_003592	Cullin-1	chr7	148395932	148498202
CUL3	NM_001257198	Cullin-3	chr2	225334866	225434542
DDB1	NM_001923	DNA damage-binding protein 1	chr11	61066918	61100684
DLL1	NM_005618	Delta-like protein 1	chr6	170591293	170599697
DYRK1A	NM_001396	Dual specificity tyrosine-phosphorylation-regulated kinase 1A	chr21	38792601	38887679
EP300	NM_001429	Histone acetyltransferase p300	chr22	41488613	41576081
EPHB2	NM_004442	Ephrin type-B receptor 2	chr1	23037330	23241823
GORASP2	NM_015530	Golgi reassembly-stacking protein 2	chr2	171785035	171823643
GPS1	NM_212492	COP9 signalosome complex subunit 1	chr17	80009762	80015346
HDAC2	NM_001527	Histone deacetylase 2	chr6	114257319	114292359
HSPA4	NM_002154	Heat shock 70 kDa protein 4	chr5	132387661	132440709
MAP3K1	NM_005921	Mitogen-activated protein kinase kinase kinase 1	chr5	56110899	56191978
MDM2	NM_002392	E3 ubiquitin-protein ligase Mdm2	chr12	69201970	69239320
MECP2	NM_001110792	Methyl-CpG-binding protein 2	chrX	153295685	153363188
MYC	NM_002467	Myc proto-oncogene protein	chr8	128748314	128753680
NCOR1	NM_006311	Nuclear receptor corepressor 1	chr17	15933407	16118874
PBRM1	NM_018313	Protein polybromo-1	chr3	52579367	52719866
PIAS1	NM_016166	E3 SUMO-protein ligase PIAS1	chr15	68346571	68480404
PSMA7	NM_002792	Proteasome subunit alpha type-7	chr20	60711782	60718514
RAB2A	NM_002865	Ras-related protein Rab-2A	chr8	61429468	61536203
RB1	NM_000321	Retinoblastoma-associated protein	chr13	48877882	49056026
RUVBL1	NM_003707	RuvB-like 1	chr3	127799799	127842671
SETD5	NM_001080517	SET domain-containing protein 5	chr3	9439402	9519838
SFPQ	NM_005066	Splicing factor, proline- and glutamine-rich	chr1	35649200	35658743
SMAD2	NM_005901	Mothers against decapentaplegic homolog 2	chr18	45359465	45456970
SMARCC2	NM_003075	SWI/SNF complex subunit SMARCC2	chr12	56555635	56583351
SMARCC1	NM_003074	SWI/SNF complex subunit SMARCC1	chr3	47627377	47823405
SMC3	NM_005445	Structural maintenance of chromosomes protein 3	chr10	112327448	112364392
STAG1	NM_005862	Cohesin subunit SA-1	chr3	136055998	136471245
SUMO2	NM_006937	Small ubiquitin-related modifier 2	chr17	73163824	73179098
TBR1	NM_006593	T-box brain protein 1	chr2	162272619	162281573
TCF3	NM_003200	Transcription factor E2-alpha	chr19	1609288	1652328
TCF4	NM_001243226	Transcription factor 4	chr18	52889561	53303188
TRAF2	NM_021138	TNF receptor-associated factor 2	chr9	139780964	139821067
TRRAP	NM_001244580	Transformation/transcription domain-associated protein	chr7	98476112	98610866
TUBA1A	NM_006009	Tubulin alpha-1A chain	chr12	49578577	49583107
UIMC1	NM_001199298	BRCA1-A complex subunit RAP80	chr5	176332005	176433795
VCP	NM_007126	Transitional endoplasmic reticulum ATPase	chr9	35056064	35072739
WDR5	NM_017588	WD repeat-containing protein 5	chr9	137001209	137025094
YY1	NM_003403	Transcriptional repressor protein YY1	chr14	100705101	100744804
ZMYND11	NM_006624	Zinc finger MYND domain-containing protein 11	chr10	180404	300577
M2					

CALM1	NM_006888	Calmodulin	chr14	90863326	90874619
DLG3	NM_021120	Disks large homolog 3	chrX	69664704	69725339
DLG4	NM_001365	Disks large homolog 4	chr17	7093209	7123369
DLGAP1	NM_004746	Disks large-associated protein 1	chr18	3496029	4455266
DUSP3	NM_004090	Dual specificity protein phosphatase 3	chr17	41843488	41856368
GRIN2A	NM_000833	Glutamate receptor ionotropic, NMDA 2A	chr16	9847264	10276611
GRIN2B	NM_000834	Glutamate receptor ionotropic, NMDA 2B	chr12	13714409	14133022
HTR2A	NM_000621	5-hydroxytryptamine receptor 2A	chr13	47405676	47471211
KCNB1	NM_004975	Potassium voltage-gated channel subfamily B member 1	chr20	47988504	48099181
KCNH1	NM_172362	Potassium voltage-gated channel subfamily H member 1	chr1	210851656	211307457
KCNMA1	NM_001161352	Calcium-activated potassium channel subunit alpha-1	chr10	78644634	79397577
MAP1A	NM_002373	Microtubule-associated protein 1A	chr15	43809805	43823818
MAPK1	NM_002745	Mitogen-activated protein kinase 1	chr22	22113946	22221970
RIMS1	NM_014989	Regulating synaptic membrane exocytosis protein 1	chr6	72596405	73112845
RPS6KA3	NM_004586	Ribosomal protein S6 kinase alpha-3	chrX	20168028	20284750
SHANK2	NM_012309	SH3 and multiple ankyrin repeat domains protein 2	chr11	70313960	70935808
STX1A	NM_004603	Syntaxin-1A	chr7	73113534	73134017
STXBP1	NM_003165	Syntaxin-binding protein 1	chr9	130374485	130454995
SV2B	NM_014848	Synaptic vesicle glycoprotein 2B	chr15	91643538	91844539
SYNGAP1	NM_006772	Ras/Rap GTPase-activating protein SynGAP	chr6	33387846	33421466
SYT1	NM_001135806	Synaptotagmin-1	chr12	79439432	79845788

Note:
Positions refers to GRCh37/hg19 assembly.

Supplementary Table S2. List of Species.

Vertebrata	
**Common Name**	***Scientific name***
Mammalia	
Human	*Homo sapiens*
Chimpanzee	*Pan troglodytes*
Gorilla	*Gorilla gorilla gorilla*
Orangutan	*Pongo abelii*
Macaque	*Macaca mulatta*
Marmoset	*Callithrix jacchus*
Tree Shrew	*Tupaia belangeri*
Mouse	*Mus musculus*
Rat	*Rattus norvegicus*
Pig	*Sus scrofa*
Dolphin	*Tursiops truncatus*
Cow	*Bos taurus*
Cat	*Felis catus*
Dog	*Canis lupus familiaris*
Megabat	*Pteropus vampyrus*
Hedgehog	*Erinaceus europaeus*
Elephant	*Loxodonta africana*
Opossum	*Monodelphis domestica*
Platypus	*Ornithorhynchus anatinus*
Sauropsida	
Peregrine Falcon	*Falco peregrinus*
Zebra Finch	*Taeniopygia guttata*
Sparrow	*Zonotrichia albicollis*
Budgerigar	*Melopsittacus undulatus*
Pigeon	*Columba livia*
Chicken	*Gallus gallus*
American Alligator	*Alligator Mississippiensis*
Chinese softshell turtle	*Pelodiscus sinensis*
Anole lizard	*Anolis carolinensis*
Fishes	
Coelacanth	*Latimeria chalumnae*
Fugu	*Takifugu rubripes*
Medaka	*Oryzias latipes*
Zebrafish	*Danio rerio*
Lamprey	*Petromyzon marinus*

Mammals (Extended phylogeny)	
**Common Name**	***Scientific name***
Human	*Homo sapiens*
Chimpanzee	*Pan troglodites*
Gorilla	*Gorilla gorilla gorilla*
Orangutan	*Pongo pygmaeus abelii*

Gibbon	*Nomascus leucogenys*
Baboon	*Papio hamadryas*
Mandrill	*Mandrillus leucophaeus*
Rhesus macaque	*Macaca mulatta*
Crab-eating macaque	*Macaca fascicularis*
Green monkey	*Chlorocebus sabaeus*
Snub-nosed monkey	*Rhinopithecus roxellana*
Sclater's Angola colobus	*Colobus angolensis palliatus*
Squirrel monkey	*Saimiri boliviensis*
Nancy Ma's night monkey	*Aotus nancymaae*
Marmoset	*Callithrix jacchus*
Coquerel's sifaka	*Propithecus coquereli*
Bushbaby	*Otolemur garnettii*
Chinese tree shrew	*Tupaia chinensis*
Mouse	*Mus musculus*
Rat	*Rattus norvegicus*
Chinese hamster	*Cricetulus griseus*
Golden hamster	*Mesocricetus auratus*
Prairie vole	*Microtus ochrogaster*
Lesser Egyptian jerboa	*Jaculus jaculus*
Squirrel	*Spermophilus tridecemlineatus*
Guinea pig	*Cavia porcellus*
Chinchilla	*Chinchilla lanigera*
Brush-tailed rat	*Octodon degus*
Naked mole-rat	*Heterocephalus glaber*
Rabbit	*Oryctolagus cuniculus*
Pika	*Ochotona princeps*
Cow	*Bos taurus*
Tibetan antelope	*Pantholops hodgsonii*
Sheep	*Ovis aries*
Domestic goat	*Capra hircus*
Dolphin	*Tursiops truncatus*
Killer whale	*Orcinus orca*
Alpaca	*Vicugna pacos*
Bactrian camel	*Camelus ferus*
Pig	*Sus scrofa*
Pacific walrus	*Odobenus rosmarus divergens*
Weddell seal	*Leptonychotes weddellii*
Panda	*Ailuropoda melanoleuca*
Ferret	*Mustela putorius furo*
Dog	*Canis lupus familiaris*
Cat	*Felis catus*
Horse	*Equus caballus*
White rhinoceros	*Ceratotherium simum*
Black flying-fox	*Pteropus alecto*

Megabat	*Pteropus vampyrus*
David's myotis bat	*Myotis davidii*
Microbat	*Myotis lucifugus*
Big brown bat	*Eptesicus fuscus*
Hedgehog	*Erinaceus europaeus*
Shrew	*Sorex araneus*
Star-nosed mole	*Condylura cristata*
Elephant	*Loxodonta africana*
Cape elephant shrew	*Elephantulus edwardii*
Manatee	*Trichechus manatus latirostris*
Cape golden mole	*Chrysochloris asiatica*
Tenrec	*Echinops telfairi*
Aardvark	*Orycteropus afer afer*
Armadillo	*Dasypus novemcinctus*

Supplementary Table S3. Likelihood ratio test statistics for models of variable selective pressure among branches (codon frequency:F61).

	Genes	Mammalian branch		Sauropsidan branch	
		**− 2ΔlnL**[a]	***p* value**[b]	**− 2ΔlnL**[a]	***p* value**[b]
M1	***CHD8***	54.380	1.65×10^{-13}	95.668	2.72×10^{-22}
	HSPA4	0	1	11.213	1.62×10^{-4}
	MDM2	7.120	1.53×10^{-2}	0	1
	MECP2	4.612	3.17×10^{-2}	21.943	5.62×10^{-6}
	MYC	5.139	4.68×10^{-2}	2.058	1.51
	RB1	0	1	10.725	$\times 10^{-3}$
	SMARCC2 Region 2 (106-1152aa)	244.527	8.10×10^{-55}	0	1
	UIMC1	7.465	1.26×10^{-2}	5.898	1.52×10^{-2}
M2	***DLG4***	9.006	5.38×10^{-3}	0	1
	GRIN2A Region 3 (867-1464aa)	5.670	3.45×10^{-2}	0	1
	GRIN2B	0	1	8.387	7.56×10^{-3}
	KCNMA1	17.807	2.44×10^{-5}	18.811	2.44×10^{-5}
	MAP1A	81.384	3.72×10^{-19}	36.117	1.86×10^{-9}
	SYNGAP1 Region 2 (115-1343aa)	80.839	4.90×10^{-19}	31.782	1.72×10^{-8}

Notes:
a. 2ΔlnL: twice the difference of the natural logs of the maximum likelihood of the models being compared.
b. *p* values are FDR corrected.

Supplementary Table S4. Likelihood ratio test (LRT) statistics for models of variable selective pressure among sites in mammalian phylogeny (codon frequency: F61).

		M7 vs M8		M8a vs M8	
		**− 2ΔlnL**[a]	***p* value**[b]	**− 2ΔlnL**[a]	***p* value**[b]
M1	***MDM2***	24.023	1.215×10^{-5}	4.953	2.605×10^{-2}
	MECP2 (Reg1)	13.373	2.496×10^{-3}	6.108	1.346×10^{-2}
	MYC	6.742	3.435×10^{-2}	7.626	1.151×10^{-2}
	SMARCC2	51.739	1.164×10^{-11}	13.461	2.436×10^{-4}
	UIMC1	113.390	4.773×10^{-25}	67.351	2.272×10^{-16}
M2	***GRIN2A***	86.066	4.092×10^{-19}	42.737	6.262×10^{-11}
	MAP1A	90.533	4.384×10^{-20}	72.934	1.334×10^{-17}
	SYNGAP1 (Reg1)	38.285	9.719×10^{-9}	15.875	6.765×10^{-5}

Notes:
Models: M7 is a null model that assumes that $0<\omega<1$ is beta distributed among sites; M8 (positive selection model) is the same as M7 but also includes an extra category of sites with $\omega>1$. M8a is the same as M8, except that the 11th category cannot allow positive selection, but only neutral evolution.
a. 2ΔlnL: twice the difference of the natural logs of the maximum likelihood of the models being compared.
b. *p* values are FDR corrected.

Supplementary Table S5. *f*-values and Introgression scores.

Group	Gene	Transcript ID	chr	start	end	Introgression score (EUR)	Introgression score (ASN)	*f* value (SniPRE)
M1	*ABL1*	NM_007313	chr9	133589267	133763062	0.0066	0.0090	0.4349
M1	*ARID1B*	NM_020732	chr6	157099063	157531913	0.0333	0.0178	0.2417
M1	*CASK*	NM_003688	chrX	41374188	41782287	0.0046	0.0101	0.1928
M1	*CHD8*	NM_001170629	chr14	21853352	21899867	0.0020	0.0030	0.5521
M1	*CREB1*	NM_134442	chr2	208394615	208470284	0.0010	0.0042	0.3510
M1	*CTNNB1*	NM_001904	chr3	41240941	41281939	0.0050	0.0074	0.3078
M1	*CUL1*	NM_003592	chr7	148395932	148498202	0.0025	0.0036	0.2826
M1	*CUL3*	NM_001257198	chr2	225334866	225434542	0.0000	0.0000	0.3711
M1	*DDB1*	NM_001923	chr11	61066918	61100684	0.0080	0.0030	0.1748
M1	*DLL1*	NM_005618	chr6	170591293	170599697	0.0175	0.0734	0.4984
M1	*DYRK1A*	NM_001396	chr21	38792601	38887679	0.0556	0.0005	0.5645
M1	*EP300*	NM_001429	chr22	41488613	41576081	0.0000	0.0000	0.2211
M1	*EPHB2*	NM_004442	chr1	23037330	23241823	0.0306	0.0291	0.2426
M1	*GORASP2*	NM_015530	chr2	171785035	171823643	3.62e-05	7.00e-05	0.5701
M1	*GPS1*	NM_212492	chr17	80009762	80015346	0.0123	0.0149	0.2281
M1	*HDAC2*	NM_001527	chr6	114257319	114292359	0.0020	0.0020	0.3875
M1	*HSPA4*	NM_002154	chr5	132387661	132440709	0.0229	0.0011	0.6247
M1	*MAP3K1*	NM_005921	chr5	56110899	56191978	0.0822	0.2306	0.3505
M1	*MDM2*	NM_002392	chr12	69201970	69239320	0.0358	0.0190	0.4046
M1	*MECP2*	NM_001110792	chrX	153295685	153363188	0.0133	0.0150	0.4712
M1	*MYC*	NM_002467	chr8	128748314	128753680	0.0808	0.1252	0.5125
M1	*NCOR1*	NM_006311	chr17	15933407	16118874	5.20e-05	9.00e-05	0.2989
M1	*PBRM1*	NM_018313	chr3	52579367	52719866	0.0000	0.0030	0.4482
M1	*PIAS1*	NM_016166	chr15	68346571	68480404	0.0016	0.0078	0.2928
M1	*PSMA7*	NM_002792	chr20	60711782	60718514	0.0010	0.0020	0.2792
M1	*RAB2A*	NM_002865	chr8	61429468	61536203	1.75e-05	0.0007	0.4015
M1	*RB1*	NM_000321	chr13	48877882	49056026	0.0011	0.0002	0.5220
M1	*RUVBL1*	NM_003707	chr3	127799799	127842671	0.0000	0.0000	0.3155
M1	*SETD5*	NM_001080517	chr3	9439402	9519838	0.0015	0.0084	0.3544
M1	*SFPQ*	NM_005066	chr1	35649200	35658743	0.0051	0.0090	0.2233
M1	*SMAD2*	NM_005901	chr18	45359465	45456970	0.0022	0.0040	0.3317
M1	*SMARCC2*	NM_003075	chr12	56555635	56583351	0.0223	0.0260	0.2056
M1	*SMARCC1*	NM_003074	chr3	47627377	47823405	0.0000	0.0000	0.4295
M1	*SMC3*	NM_005445	chr10	112327448	112364392	0.0131	0.0383	0.2632
M1	*STAG1*	NM_005862	chr3	136055998	136471245	0.0016	8.07e-06	0.2708
M1	*SUMO2*	NM_006937	chr17	73163824	73179098	0.0270	0.0110	0.4468
M1	*TBR1*	NM_006593	chr2	162272619	162281573	0.0827	0.1884	0.2999
M1	*TCF3*	NM_003200	chr19	1609288	1652328	0.0485	0.0314	0.4388
M1	*TCF4*	NM_001243226	chr18	52889561	53303188	0.0264	0.0050	0.3416
M1	*TRAF2*	NM_021138	chr9	139780964	139821067	0.0010	0.0005	0.4167
M1	*TRRAP*	NM_001244580	chr7	98476112	98610866	0.0011	0.0001	0.2618
M1	*TUBA1A*	NM_006009	chr12	49578577	49583107	0.0100	0.0080	0.3132
M1	*UIMC1*	NM_001199298	chr5	176332005	176433795	0.0010	0.0003	0.5258
M1	*VCP*	NM_007126	chr9	35056064	35072739	0.0030	0.0030	0.3697
M1	*WDR5*	NM_017588	chr9	137001209	137025094	0.0751	0.1511	0.2338
M1	*YY1*	NM_003403	chr14	100705101	100744804	0.0434	0.0222	0.2986
M1	*ZMYND11*	NM_006624	chr10	180404	300577	0.0031	0.0006	0.3189

M2	*CALM1*	NM_006888	chr14	90863326	90874619	0.0066	0.0052	0.3845
M2	*DLG3*	NM_021120	chrX	69664704	69725339	0.0026	0.0030	0.2480
M2	*DLG4*	NM_001365	chr17	7093209	7123369	0.0189	0.0243	0.3028
M2	*DLGAP1*	NM_004746	chr18	3496029	4455266	0.0379	0.0439	0.2528
M2	*DUSP3*	NM_004090	chr17	41843488	41856368	0.0021	0.0030	0.4032
M2	*GRIN2A*	NM_000833	chr16	9847264	10276611	0.0025	0.0031	0.4650
M2	*GRIN2B*	NM_000834	chr12	13714409	14133022	0.1145	0.0163	0.2694
M2	*HTR2A*	NM_000621	chr13	47405676	47471211	0.0448	0.0331	0.5698
M2	*KCNB1*	NM_004975	chr20	47988504	48099181	0.0082	0.0150	0.5409
M2	*KCNH1*	NM_172362	chr1	210851656	211307457	0.1643	0.0608	0.2738
M2	*KCNMA1*	NM_001161352	chr10	78644634	79397577	0.0311	0.0287	0.2240
M2	*MAP1A*	NM_002373	chr15	43809805	43823818	0.0000	0.0000	0.5275
M2	*MAPK1*	NM_002745	chr22	22113946	22221970	0.0400	0.2485	0.3764
M2	*RIMS1*	NM_014989	chr6	72596405	73112845	0.0140	0.0033	0.3527
M2	*RPS6KA3*	NM_004586	chrX	20168028	20284750	0.0102	0.0119	0.2627
M2	*SHANK2*	NM_012309	chr11	70313960	70935808	0.0213	0.0408	0.2673
M2	*STX1A*	NM_004603	chr7	73113534	73134017	0.0212	0.0265	0.2765
M2	*STXBP1*	NM_003165	chr9	130374485	130454995	0.0022	0.0554	0.1907
M2	*SV2B*	NM_014848	chr15	91643538	91844539	0.0134	0.0171	0.3180
M2	*SYNGAP1*	NM_006772	chr6	33387846	33421466	0.1251	0.0256	0.2169
M2	*SYT1*	NM_001135806	chr12	79439432	79845788	0.0029	0.0031	0.3479

Notes: Positions refers to GRCh37/hg19 assembly

Supplementary Table S6. Selected SNPs in early modern human populations.

Gene	SNP	Genomic Position (hg19)	Derived allele frequency[a]		
			AFR	EUR	EAS
DYRK1A	rs17814633	chr21:38769655-38769656	0.926	0.899	1
	rs11088389	chr21:38759503-38759504	0.926	0.897	1
	rs56218358	chr21:38782077-38782078	0.996	0.899	1
	rs41483945	chr21:38764243-38764244	0.926	0.897	1
	rs73216405	chr21:38750067-38750068	0.996	0.899	1
	rs17814651	chr21:38774564-38774565	0.996	0.899	1
	rs73218417	chr21:38804881-38804882	0.996	0.899	1
	rs11701543	chr21:38755882-38755883	0.927	0.899	1
PIAS1	rs2130270	chr15:68354118-68354119	0.888	0.994	1
	rs141697106	chr15:68362739-68362740	1	0.994	1
STAG1	rs111561104	chr3:136393277-136393278	0.999	1	1
TCF4	rs75595647	chr18:52971018-52971019	0.918	0.966	0.994
	rs78196382	chr18:52995852-52995853	0.847	0.961	0.994
	rs144130249	chr18:53026693-53026694	0.998	0.967	0.998
	rs79118099	chr18:53009097-53009098	0.831	0.938	0.994
	rs79034601	chr18:52997245-52997246	0.903	0.966	0.998
	rs73490861	chr18:53034300-53034301	0.966	0.966	0.998
	rs73492933	chr18:53044947-53044948	0.833	0.963	0.994
	rs116671631	chr18:53012154-53012155	0.931	0.967	0.998
	rs116342691	chr18:52999869-52999870	0.931	0.967	0.998
	rs41421645	chr18:53038732-53038733	0.803	0.962	0.994
	rs73490839	chr18:53022953-53022954	0.949	0.967	0.998
	rs73490856	chr18:53032685-53032686	0.948	0.966	0.998
	rs67800840	chr18:53030762-53030763	0.813	0.939	0.994
	rs77115755	chr18:53028853-53028854	0.844	0.966	0.998
	rs77452859	chr18:52988219-52988220	0.86	0.965	0.994
YY1	rs76662787	chr14:100719477-100719478	0.865	0.957	1
	rs61992940	chr14:100719517-100719518	0.794	0.957	1
	rs61992931	chr14:100711907-100711908	0.721	0.957	1

[a]Allelic frequencies refer to 1000 Genomes project Phase 3 data. AFR: African, EUR: European; EAS: East Asian.

Supplementary Figure S1. ***PIAS1*, *STAG1*, and *YY1* modern human alleles**

Modern-human-specific SNPs and their location in *PIAS1*, *STAG1*, and *YY1* are shown within the UCSC Genome Browser view. S scores are shown in red. The horizontal black line represents the 5th percentile of S score.

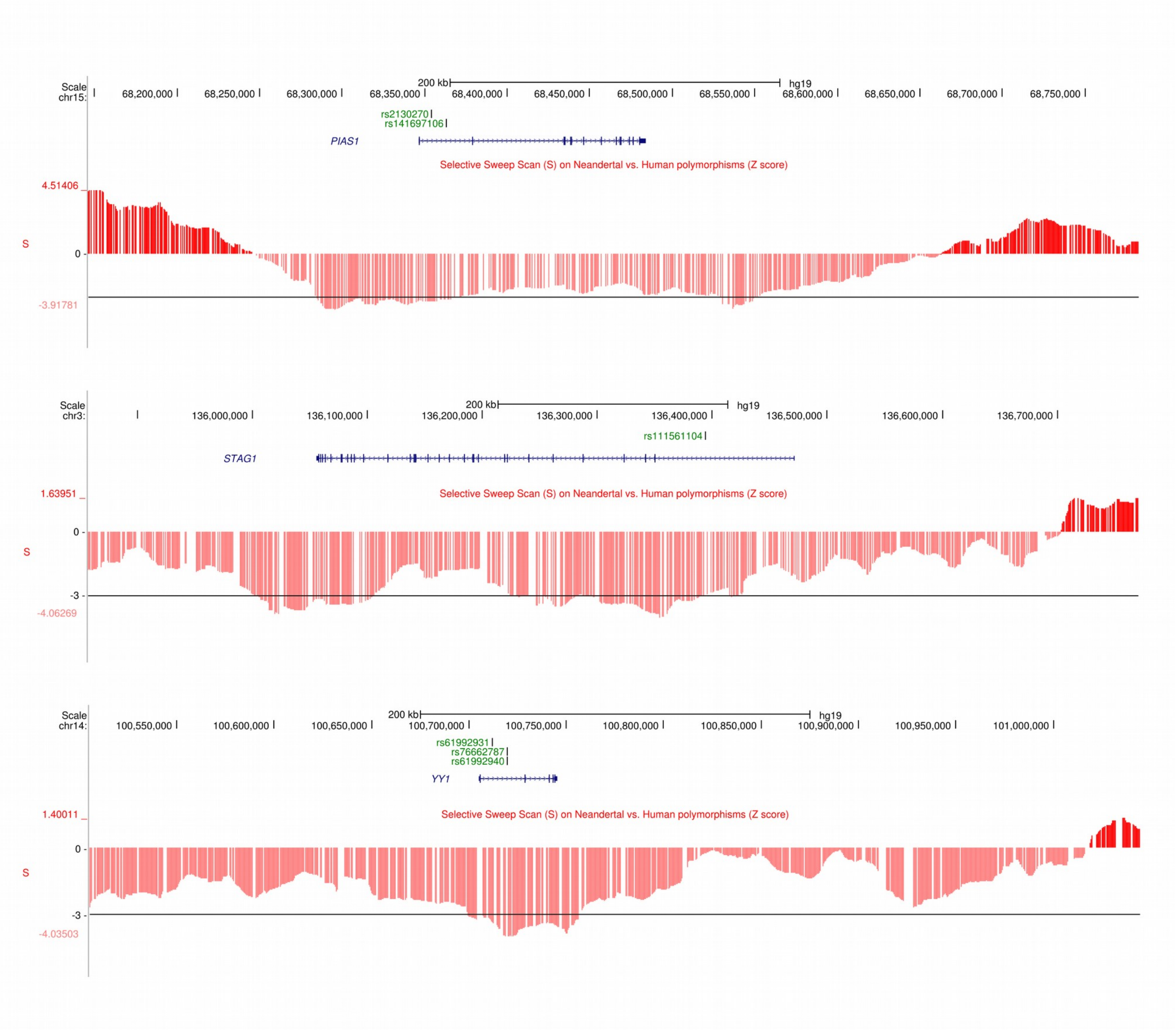

Supplement: Supplementary file 1 — Supplementary Information [file 41598_2017_6440_MOESM1_ESM.pdf]
